# Estimation of Klobuchar Coefficients for Japan and Performance Evaluation Compared with QZSS Coefficients

**DOI:** 10.3390/s26154677

**Published:** 2026-07-23

**Authors:** Ei-Ju Sim, Kwan-Dong Park

**Affiliations:** 1 Solution Development Team, PPSOL Inc., Seoul 08504, Republic of Korea; 2 Department of Geoinformatic Engineering, Inha University, Incheon 22212, Republic of Korea; kdpark@inha.ac.kr

**Keywords:** GNSS, RNSS, ionosphere, Klobuchar model, GIM

## Abstract

**Highlights:**

**What are the main findings?**

- A comparison of correction coefficients among the Klobuchar GPS (KG), Klobuchar QZSS (KQ), and Klobuchar Regional (KR) models showed that both the diversity of coefficient values and the intermodel correlations varied depending on the level of solar activity.
- The VTEC RMSE analysis at grid points over and around Japan indicated that the KR model improved accuracy by approximately 50% compared with the KQ model, achieving the best performance among the three models.

**What are the implications of the main findings?**

- The KR coefficients estimated in this study outperformed the Japan-targeted KQ model in terms of both ionospheric correction accuracy and temporal stability.
- Since the grid-point coverage can be flexibly adjusted according to the target region, the proposed methodology is expected to improve the ionospheric correction performance of the Klobuchar model in various regions.

**Abstract:**

In this study, Klobuchar coefficients optimized for the Japanese region, referred to as the Klobuchar Regional (KR) model, were estimated using the International GNSS Service (IGS) Global Ionosphere Map (GIM) and evaluated against the Klobuchar GPS (KG) and Klobuchar QZSS (KQ) models. The grid points were defined based on ionospheric pierce points (IPPs) derived from Japanese GNSS stations, and the coefficients were estimated via a nonlinear least-squares approach using the vertical total electron content (VTEC) within the defined region (12.5–52.5° N, 115–170° E) under both solar minimum (2019) and solar maximum (2024) conditions. During the solar minimum, the number of distinct KG correction coefficient values was limited to fewer than 16, and intermodel correlations were found to vary with solar activity level, with the KQ–KR pair exhibiting the highest correlation for α_0_ (*r* = 0.85). Vertical Total Electron Contents (VTEC) root mean square error (RMSE) analysis indicated that KR achieved an approximately 50% improvement in accuracy relative to both KG and KQ. Furthermore, standard point positioning (SPP) results at three Japanese stations demonstrated that KR and KQ exhibited comparable performance within 0.1 m during the solar minimum, whereas KR generally outperformed KQ during the solar maximum, with the standard deviation of KR approximately half that of KQ, confirming superior stability and reliability in positioning performance.

## 1. Introduction

In the signal propagation process of a Global Navigation Satellite System (GNSS), the ionosphere is the dominant error source for single-frequency users [1]. Dual-frequency receivers can effectively eliminate ionospheric delays by utilizing the dispersive characteristics of the ionosphere through an ionosphere-free (IF) combination [2]. However, single-frequency users rely on ionospheric correction models to mitigate these errors. Representative ionospheric correction models include the Global Ionosphere Map (GIM) and Klobuchar model. GIM is generated based on GNSS observations collected from a global network of continuously operating reference stations and provides the vertical total electron content (VTEC) in a gridded format. The model has a spatial resolution of 2.5° latitude and 5° longitude and a temporal resolution of 2 h [3]. Its accuracy is reported to be in the order of 2–8 total electron content units (TECU), making it a highly accurate ionospheric correction model. However, because GIM is a post-processed product derived from global observations, its application in real-time positioning is limited [4]. The Klobuchar model estimates ionospheric delay using eight coefficients broadcast in a global positioning system (GPS) navigation message. Because of its low computational complexity and real-time applicability, it is widely used in GNSS positioning. However, it can only correct approximately 50–60% of the ionospheric delay, resulting in relatively limited accuracy [5].

The limitations of the Klobuchar model stem from approximating the complex spatiotemporal variability of the ionosphere using a limited number of parameters and a simplified functional form, and from its reliance on only eight correction coefficients, which reduce ionospheric behavior to a highly constrained form [6]. The Klobuchar model employs a single set of coefficients broadcast globally, once daily [7]. An analysis of the coefficients collected over a 22-year period from 2002 to 2022 revealed that while some parameters exhibit variability associated with solar activity, others tend to remain nearly constant over several days [8]. This behavior indicates that short-term and regional ionospheric variations were not adequately represented, leading to degraded correction performance of the Klobuchar model.

Regional navigation systems provide their own ionospheric correction coefficients to overcome the limitations of the globally broadcast Klobuchar coefficients. The BeiDou Navigation Satellite System (BDS) adopts a modified Klobuchar model and provides correction coefficients at 2 h intervals, thereby allowing for a more effective representation of ionospheric variability [9,10]. Numerous studies have investigated the performance and improvements in Klobuchar-based ionospheric correction models [11,12,13,14]. Similarly, the Quasi-Zenith Satellite System (QZSS) broadcasts its own ionospheric correction coefficient. In the QZSS, the coefficients are divided into wide-area and Japan-area [15]. The Japan-area coefficients are applicable within the regions 22–50° N and 110–160° E, whereas the wide-area coefficients cover 60° S–60° N and 90°–180° E. Wide-area coefficients are designed for applications over a broad region encompassing Japan and Australia, whereas Japan-area coefficients are regionally optimized for users in Japan and its surrounding areas. However, the regional coefficients provided by the QZSS exhibit lower accuracy than the GPS coefficients; only a few related studies have been conducted [16].

Accordingly, this study estimated Klobuchar coefficients specifically optimized for ionospheric correction in the Japanese region using only International GNSS Service (IGS) GIM data based on the methodology proposed by [8], and evaluated their performance. To this end, the analysis domain was defined based on ionospheric pierce points (IPPs) derived from GNSS observation stations in Japan, and GIM-based VTEC values at 204 grid points within a latitude range of 12.5–52.5° N and longitude range of 115–170° E were used as reference values. The estimated coefficients were compared with the regional correction coefficients for Japan provided by the QZSS and GPS Klobuchar correction coefficients, and the ionospheric modeling performance was assessed by comparing the grid-point VTEC values computed using each set of coefficients. In addition, standard point positioning (SPP) was performed at three stations in Japan to quantitatively evaluate the positioning performance in terms of actual positioning accuracy. The remainder of this paper is organized as follows. Section 2.1 provides a brief overview of the ionospheric models used for coefficient estimation: GIM and Klobuchar model. Section 2.2 presents the methodology for estimating the correction coefficients using observations from each model, and Section 2.3 describes the grid region and datasets used in this study. Section 3.1 analyzes the temporal characteristics of the coefficients during the solar minimum (2019) and solar maximum (2024), while Section 3.2 evaluates the accuracy of VTEC derived from each set of coefficients. Finally, in Section 3.3, the positioning results from three stations in Japan were compared and analyzed over the analysis period to validate the performance and applicability of the proposed coefficients.

## 2. Method and Data

Section 2.1 briefly describes the ionospheric correction models used in this study, namely Klobuchar model and the GIM. Section 2.2 presents the methodology for estimating correction coefficients optimized for the Japanese region, while Section 2.3 details the data configuration and experimental setup employed in the analysis.

### 2.1. Ionospheric Model

The Klobuchar model assumes that ionospheric electrons are concentrated in a single thin layer located approximately 350 km above the Earth’s surface. The ionospheric delay was modeled as a constant value of 5 ns during the nighttime and as a half-cosine function centered at 14:00 local time during the daytime (Equation (1)).

(1) $$T_{iono}=F\left[DC+AMP\left(1-\frac{x^{2}}{4}+\frac{x^{4}}{24}\right)\right]$$

In Equation (1), *F* denotes the mapping function used to convert VTEC into Slant Total Electron Content (STEC). *DC* represents the nighttime delay constant of 5 ns, *AMP* denotes the amplitude of the half-cosine function, and *x* represents the phase expressed in radians.

(2) $$AMP=\left\{\begin{array}{cc} \sum_{n=0}^{3}\alpha_{n}\phi_{m}^{n}, & AMP\geq 0\\ 0, & AMP<0 \end{array}\right.$$

(3) $$PER=\left\{\begin{array}{cc} \sum_{n=0}^{3}\beta_{n}\phi_{m}^{n}, & PER\geq 72000\\ 72000, & PER<72000 \end{array}\right.$$

Here, the amplitude (AMP) and period (PER) of the half-cosine function are computed from the alpha and beta coefficients broadcast in the navigation message, respectively, using Equations (2) and (3). Specifically, AMP and PER are expressed as polynomial functions of the four alpha and four beta coefficients, $\alpha_{n}(n=0,1,2,3)$ and $\beta_{n}(n=0,1,2,3)$, respectively, with respect to the geomagnetic latitude of the IPP, denoted as $\phi_{m}$.

According to Equation (3), the width of the daytime interval over which the AMP term is applied corresponds to half of the period. Accordingly, the lower bound of PER is set to 72,000 s (20 h), thereby ensuring that the daytime ionospheric delay is maintained over a minimum duration. Finally, the vertical ionospheric delay is computed using the receiver and satellite positions, as well as the eight Klobuchar coefficients broadcasted in the navigation message. Further details of the procedure are provided in [5].

GIM are grid-based ionospheric models generated from observations collected at GNSS stations worldwide. GIM products are currently provided by several Ionosphere Associate Analysis Centers (IAACs), including Center for Orbit Determination in Europe (CODE), Chinese Academy of Sciences (CAS), and Jet Propulsion Laboratory (JPL). In particular, the IGS final and rapid GIM products are generated by combining products from several IAACs using a weighted averaging method [17]. They assumed the ionosphere to be a single-layer shell at an altitude of approximately 450 km and provided VTEC values at each grid point [18]. The VTEC was computed by estimating the spherical harmonic coefficients from dual-frequency observations collected across a global network of stations, which were then used to generate the global VTEC distribution. To utilize GIM, users must compute the IPP between the receiver and satellite and interpolate the VTEC from the four adjacent grid points. In this process, temporal interpolation accounts for GIM’s 2 h intervals using spline interpolation, whereas spatial interpolation is performed via bicubic interpolation. The final interpolated VTEC was then converted into STEC using a mapping function for ionospheric delay correction. In this study, GIM VTEC was used as the reference value for estimating the Klobuchar coefficients using a least-squares approach. Cubic spline interpolation was applied in the temporal domain, whereas bilinear interpolation using the four surrounding grid points at the IPP was performed in the spatial domain [3].

### 2.2. Nonlinear Least-Squares Estimation

Nonlinear least-squares estimation (NLSE) was applied to estimate the Klobuchar coefficients. NLSE is a method for estimating unknown parameters by minimizing the differences between the observed values and model predictions [19]. In this study, the coefficients were estimated by minimizing the differences between the ionospheric delays derived from the GIM and those computed using the Klobuchar model. The nonlinear least-squares problem can be expressed as follows.

(4) $$J\hat{x}=K$$

where *J* denotes the Jacobian matrix, which is composed of the partial derivatives of the Klobuchar model with respect to the eight coefficients ($\alpha_{0}$*~*$\alpha_{3}$*,* $\beta_{0}$*~*$\beta_{3}$). Matrix *J* has dimensions of *n* × 8, where *n* denotes the total number of VTEC observations generated at 2 h intervals for all grid points defined over the Japanese region. The partial derivative expressions for each coefficient are derived from the nonlinear model structure, and their detailed formulations are provided by [8]. *K* denotes the residual vector, defined as the difference between the VTEC values derived from the IGS final GIM and those computed using the Klobuchar model over the Japanese region. However, it should be noted that the GIM used as the observation data in this process assumes a single-layer height of 450 km, whereas the Klobuchar model assumes a single-layer height of 350 km. Consequently, errors may be introduced due to the difference in the assumed single-layer height between the two models. $\hat{x}$ represents the increment vector of the Klobuchar coefficients. The coefficients were iteratively updated through a linearization process, with the number of iterations set to five. At each iteration, the coefficients were refined to minimize the residuals, thereby yielding the optimal set of Klobuchar coefficients. The mean values of the coefficients collected over 11 years at the continuously operating DAEJ station were used as initial values for the coefficient estimation [8].

### 2.3. Study Area and Data

**Figure 1:**
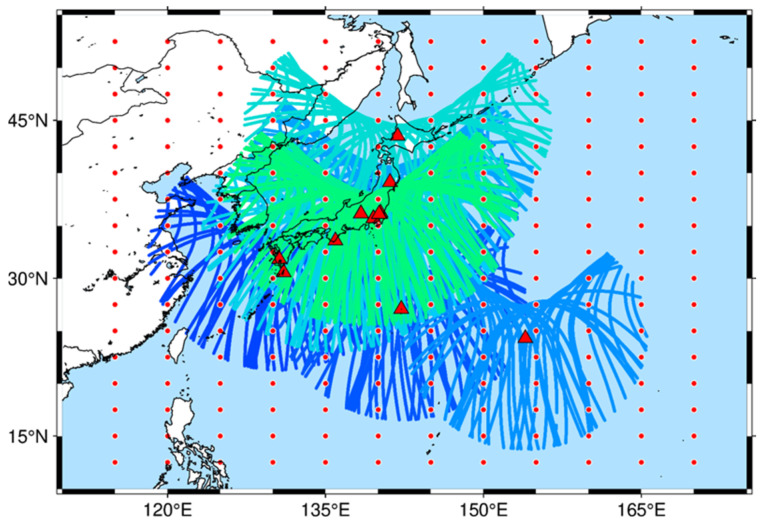
Two hundred four GIM grid points used for generating regionally optimized Klobuchar coefficients over the Japan region. The grid points used in this study are presented as red dots; the red triangles indicate the GNSS stations used for IPPs computation, and the blue lines represent the 24 h IPPs trajectories derived from each station.

To estimate the correction coefficients optimized for the Japanese region, grid points corresponding to the areas surrounding Japan were selected from the global grid and used in the analysis. To this end, 15 IGS stations in Japan were selected, and the spatial distribution of the IPPs was analyzed based on 24 h GNSS observations at each station. The resulting IPPs distribution is shown in Figure 1. Based on this distribution, the grid region was defined to cover latitudes from 12.5° N to 52.5° N at 2.5° intervals (17 points) and longitudes from 115° E to 170° E at 5° intervals (12 points), consisting of 204 grid points (17 × 12).

The Daejeon station, operated by the Korea Astronomy and Space Science Institute (KASI), provides continuous and reliable GNSS observations over extended periods. Because DAEJ is located within the defined Japan-area region (22–50° N, 110–160° E), it was selected in this study as the data collection station for acquiring the QZSS broadcast ionospheric correction coefficients used in the model comparison. Klobuchar QZSS coefficients (KQ) were derived from QZSS observation data received at the DAEJ, whereas Klobuchar GPS coefficients (KG) were extracted from the GPS navigation message. For clarity, the coefficients estimated in this study are referred to as Klobuchar Regional (KR).

To evaluate the accuracy of the estimated coefficients, two analysis periods were selected considering the solar activity cycle. As the ionosphere is strongly affected by variations in solar activity, periods with different solar conditions need to be included to reflect diverse ionospheric environments [20]. Based on the approximately 11-year solar cycle, the most recent solar cycle 25 was considered, where the solar minimum corresponds to 2019 and the solar maximum to 2024 [21]. Therefore, two representative periods corresponding to the solar minimum and maximum were selected. Throughout these periods, one set of coefficients was obtained daily for each of the three models (KG, KQ, and KR).

## 3. Results

As described in Section 2.3, the KR model estimated in this study was compared and analyzed with the KG and KQ models. First, the time series characteristics of the correction factors of the three models were analyzed to quantitatively evaluate the variability of the correction factor values according to the level of solar activity and the correlation between the correction factors of the models. Subsequently, the VTEC accuracy within the set grid range calculated by applying the three models was compared with that of the IGS GIM. Finally, to perform a verification independent of GIM, SPP was conducted for one year each during the solar minimum (2019) and solar maximum (2024) at three stations in Japan to evaluate the performance in terms of positioning accuracy.

### 3.1. Comparison of Klobuchar Coefficients

**Figure 2:**
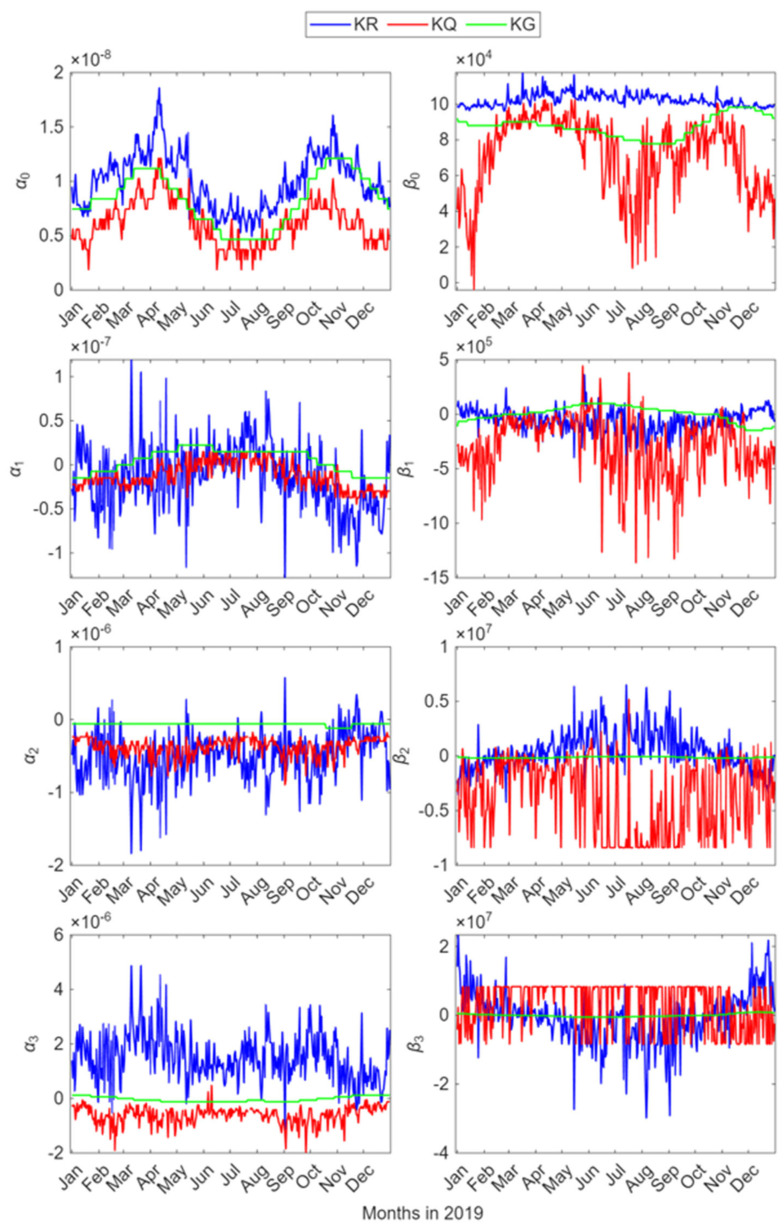
Time series of Klobuchar coefficients over one year (2019). The first col represents *α* coefficients and the second col represents *β* coefficients.

Figure 2 shows the time series variations in KG, KQ, and KR in 2019 (solar minimum period), where the first and second columns represent the *α* and *β* coefficients, respectively. The green, red, and blue lines represent KG, KQ, and KR, respectively. During the solar minimum, the KG exhibited little day-to-day variation across all correction coefficients, and the same values were often sustained on consecutive days, resulting in a very limited variety of coefficient values. In fact, even *β*_3_, which had the greatest number of unique values among KG’s correction coefficients, contained no more than 16 distinct values, a characteristic that contrasts sharply with KQ and KR, which possess more than 100 unique values. The GPS ground control station selects one from a set of predefined coefficients based on the level of solar activity and broadcasts the corresponding correction coefficients [22]. Accordingly, the number of correction coefficients decreased in 2019, the solar minimum year, owing to the subdued level of solar activity during that period. In addition to the differences in the number of unique coefficient values among the models, the KG model exhibited smaller day-to-day variability in its correction coefficients than the KQ and KR models. This result suggests that the KG model is operated more conservatively and stably, as it is intended for global broadcast.

This interpretation is consistent with the long-term behavior of the KG model reported in [8]. The 22-year time series analysis showed that the correction coefficients varied according to the solar cycle, while only a limited set of coefficient values was repeatedly used. To examine whether similar characteristics also appear in the KQ and KR models, annual correlation coefficients among the individual correction coefficients of each model were calculated, as summarized in Table 1. For *α*_0_, all three models showed a tendency for the coefficient values to increase in spring and autumn and decrease in summer and winter. This is attributed to the increased ionospheric activity in spring and autumn, which influences *α*_0_, the correction coefficient representing the amplitude of ionospheric delay. For *α*_0_, the correlation coefficients of KG–KQ, KG–KR, and KQ–KR were 0.68, 0.79, and 0.85, respectively, indicating high correlation across all three pairs. In particular, the correlation coefficient of KQ–KR was the highest, confirming that the two correction coefficients reflected ionospheric amplitude characteristics in a similar manner. For *β*_3_, the correlation coefficient of KG–KR was 0.63, indicating a positive correlation, whereas those of KG–KQ and KQ–KR were −0.41 and −0.27, respectively, indicating negative correlations. This implies that the *β*_3_ of KQ exhibits variation characteristics that are distinct from those of KG and KR. For the remaining correction coefficients, most correlation coefficients were below 0.5, and no statistically meaningful correlation was found between them, with a tendency for common variation patterns between models to diminish as the order of the coefficients increased.

**Figure 3:**
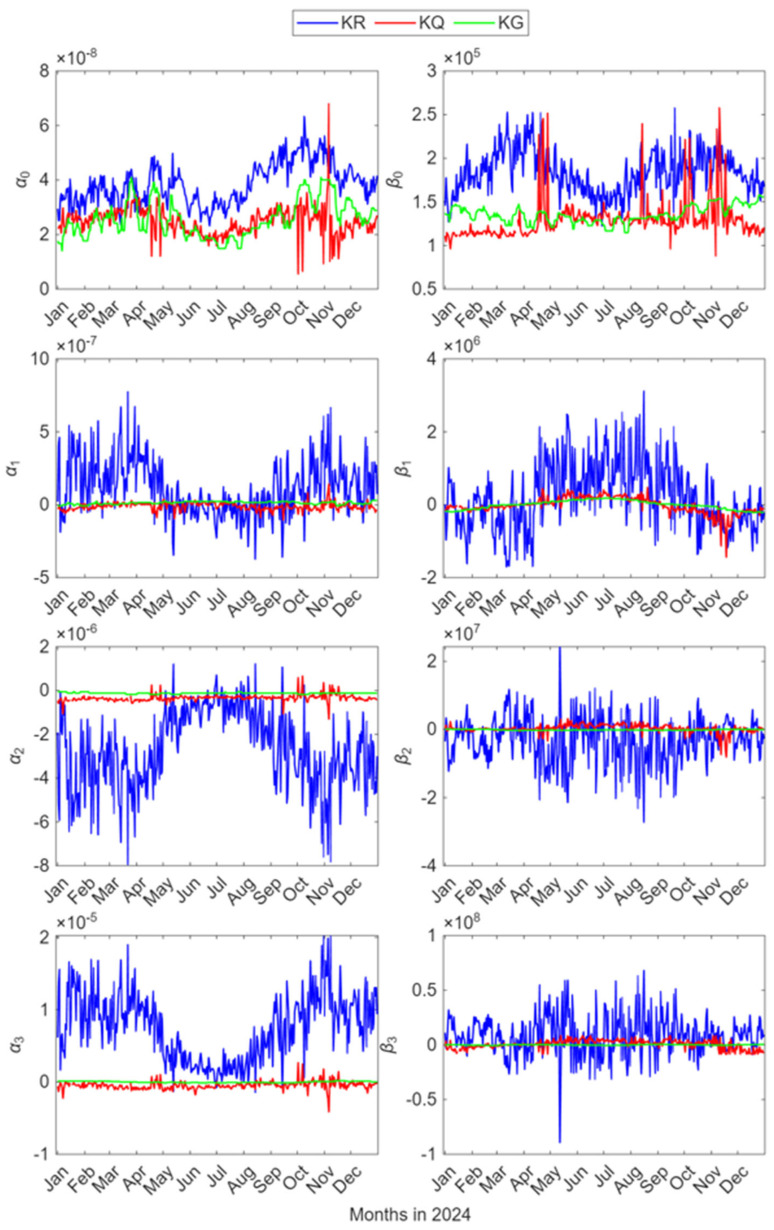
Time series of Klobuchar coefficients over one year (2024). The first col represents *α* coefficients and the second col represents *β* coefficients.

Figure 3 presents the time series characteristics of the correction coefficients for each model in 2024 (the solar maximum period). During the solar maximum, the variety of correction coefficient values expanded across all three models compared with 2019. In 2019, during the solar minimum, the number of distinct values for KG’s correction coefficients reached a maximum of only 16 (*β*_3_); however, in 2024, during the solar maximum, this increased to a maximum of 30 (*α*_0_). This was attributed to the increased ionospheric variability accompanying the heightened solar activity, which led the GPS ground control station to broadcast a greater variety of coefficient sets than those in 2019. This finding suggests that the number of unique values of KG’s correction coefficients may serve as an indicator of the level of solar activity. Furthermore, the *β*_0_ coefficient of the KQ model exhibited a pronounced increase during specific periods, particularly in April and from August to November. These periods coincided with relatively high F10.7 values, indicating that the QZSS correction coefficients were highly sensitive to variations in solar activity.

For KR, the *α*_n_ coefficients and *β*_0_ and *β*_1_ exhibited periodic seasonal variation, whereas no distinct seasonal variability was observed in *β*_2_ and *β*_3_. The intermodel correlation coefficients calculated for 2024 revealed that, in contrast with those in 2019, the correlation coefficient of *α*_0_ between KG and KR was the highest at 0.71, while that of *α*_0_ between KQ and KR was only 0.25 (Table 2). The correlation coefficients for *β*_n_ (n = 0, 1, 2, 3) were all below 0.5, indicating that no common tendency among the models was apparent during the solar maximum period. The preceding analysis confirmed that the intermodel correlation coefficients differed between the solar minimum and solar maximum periods, thereby allowing for a quantitative understanding that the intermodel correlations vary with the level of solar activity.

### 3.2. VTEC Accuracy Assessment

In Section 3.2, the VTEC values within the defined grid domain are computed using KR, the model estimated in this study and its accuracy is subsequently evaluated. The accuracy was assessed by calculating the root mean square error (RMSE) with reference to the GIM VTEC provided by the IGS. Because the temporal resolution of GIM is 2-h intervals, KR was likewise applied at 2 h intervals to compute the VTEC in this study. Continuous VTEC time series were generated over 365 days for 2019 (solar minimum) and 2024 (solar maximum), and the RMSE relative to the GIM was calculated. In addition, the performance of KR was compared with that of the KG and KQ models.

**Figure 4:**
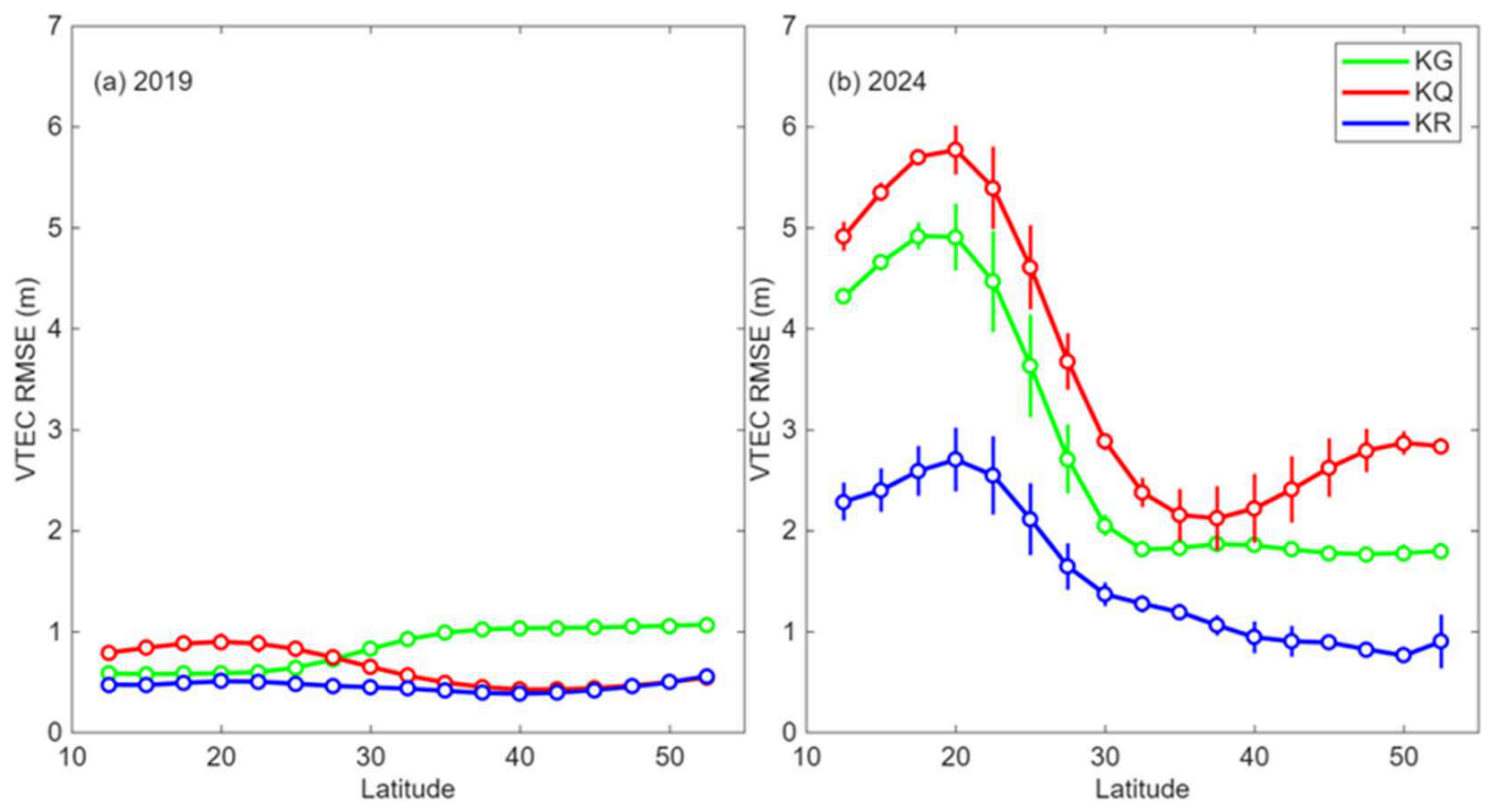
Latitudinal distribution of mean VTEC RMSE values computed from 12 grid points at 5° longitude intervals. Panel (**a**) shows results for 2019 (solar minimum), and panel (**b**) shows results for 2024 (solar maximum). Error bars represent the standard deviation at each latitude. The standard deviation ranges up to 3 cm in (**a**) and from 5 to 51 cm in (**b**). Because of their relatively small magnitudes, the error bars in (**a**) are barely visible at the scale used.

Because the ionosphere exhibits strong latitude dependence, with TEC and its variability tending to increase toward lower latitudes [23,24], a latitude-dependent accuracy analysis was conducted. The grid points used in the analysis consisted of 204 locations comprising 17 latitude bands ranging from 12.5° N to 52.5° N at 2.5° intervals, with 12 longitudinal grid points per latitude band. The mean and standard deviation of the VTEC RMSE across the 12 grid points were calculated for each latitude band, and the results are shown in Figure 4. Figure 4a and Figure 4b show the results for 2019 and 2024, respectively.

In 2019 (Figure 4a), KQ exhibited the largest variability in the low-latitude region, with the standard deviation increasing to a maximum of approximately 9 cm. In contrast, the maximum standard deviation of KR was approximately 2 cm, confirming that the RMSE was consistently computed across the latitude bands. Regarding the mean RMSE, KQ showed a tendency to increase from approximately 0.55 m at 52.5° N to approximately 0.90 m near 20° N, with errors growing toward lower latitudes. KR, however, maintained a level of approximately 0.39–0.56 m across the entire latitude range, demonstrating the most stable performance among the three models. Furthermore, the performance of KQ degraded more significantly than that of KG at low latitudes.

In 2024 (Figure 4b), all models exhibited a tendency to increase the RMSE toward lower latitudes, with the mean VTEC RMSE reaching its maximum near 20° N. KQ showed the largest error, increasing from approximately 2.84 m at 52.5° N to approximately 5.77 m at 20° N. KG increased from approximately 1.80 m to 4.91 m at the same latitudes, while KR increased from approximately 0.90 m to 2.71 m. The large errors observed around 20° N are likely associated with the Equatorial Ionization Anomaly (EIA), which extends over the latitude range of approximately 0–30° N. Since the northern EIA crest in the Northern Hemisphere is located near 20° N, all models showed consistently larger errors in this latitude band [25,26,27]. Across the entire latitude range, the maximum mean VTEC RMSE of KQ was 5.77 m, the highest among the three models, whereas the maximum of KR was 2.71 m, corresponding to approximately 50% of that value. These results demonstrate that KR maintains high accuracy and stability during the solar minimum and solar maximum periods.

### 3.3. Positioning Validation

**Figure 5:**
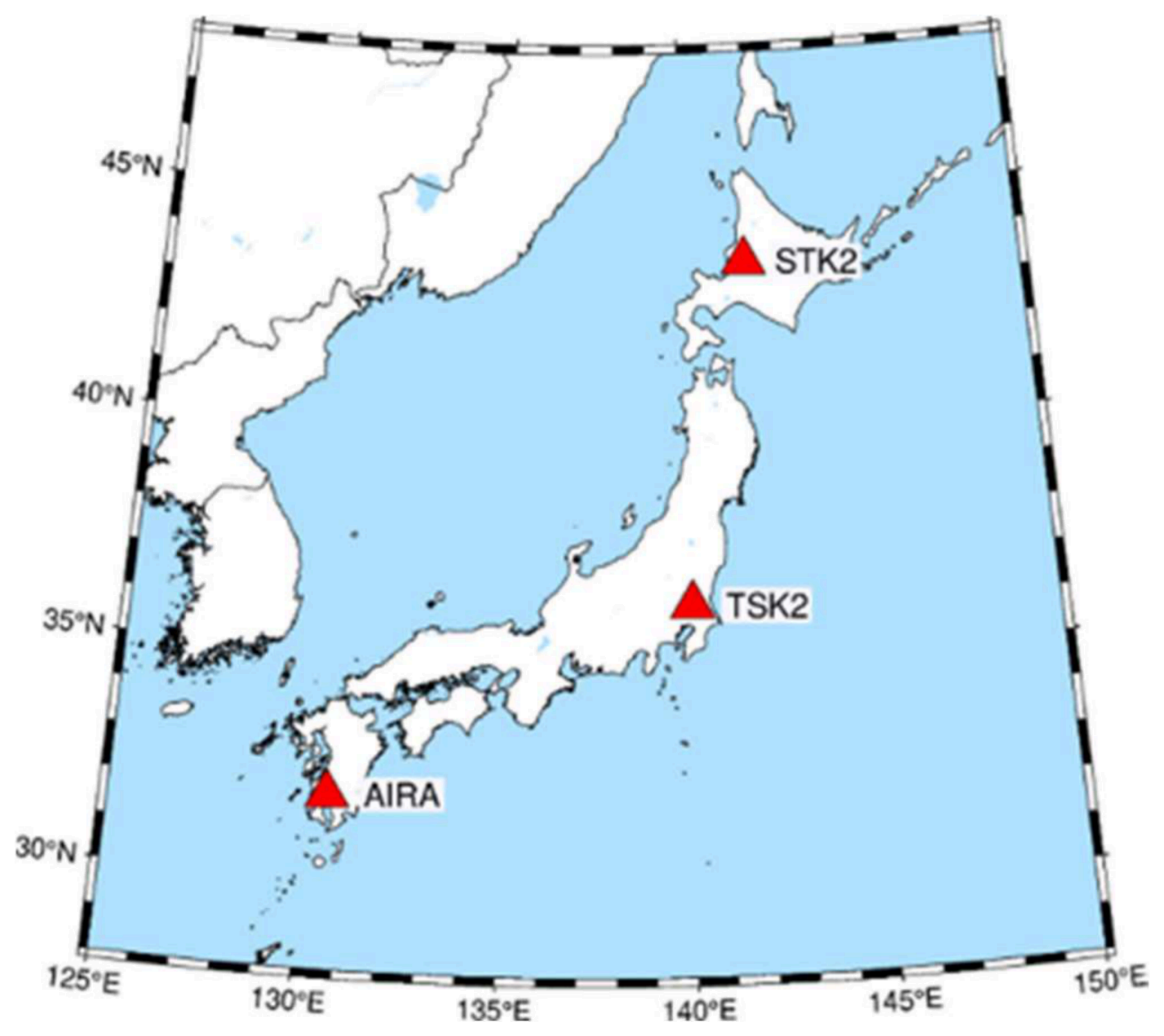
Locations of three GNSS stations in Japan selected at approximately 5° latitude intervals (STK2, TSK2, and AIRA).

In this study, the IGS GIM was simultaneously used as input data for correction coefficient estimation and as reference data for VTEC RMSE validation. Section 3.3 presents an independent validation of the KR model based on SPP using GNSS observation data acquired at three stations in Japan, rather than on IGS GIM data used for coefficient estimation. Because the positioning results derived from GNSS observations directly reflect the actual positioning environment, they serve as an effective means of evaluating the performance of each model independently of the IGS GIM. To account for latitude-dependent variations in ionospheric characteristics, three stations located in the northern, central, and southern regions of Japan were selected; their locations and geographic coordinates are shown in Figure 5. The analysis period was set to the full annual period (DOY 1–365) of 2019 (solar minimum) and 2024 (solar maximum), which was consistent with the VTEC comparative analysis.

SPP was performed daily using 24 h GNSS observation data collected at 30 s intervals at each station. The ionospheric correction was applied independently using GIM, KG, KQ, and KR. The tropospheric delay was corrected using a Global Pressure and Temperature (GPT) model, whereby the zenith delay was first estimated and then projected onto the satellite line-of-sight direction using a mapping function. Satellites with elevation angles below 15° were excluded from the observations, and those with elevation angles between 15° and 30° were down-weighted according to their elevation angle to reduce their contribution to positioning. The final position was estimated using the weighted least-squares (WLS) method, and the performance of each model was evaluated based on the three-dimensional positioning errors.

**Figure 6:**
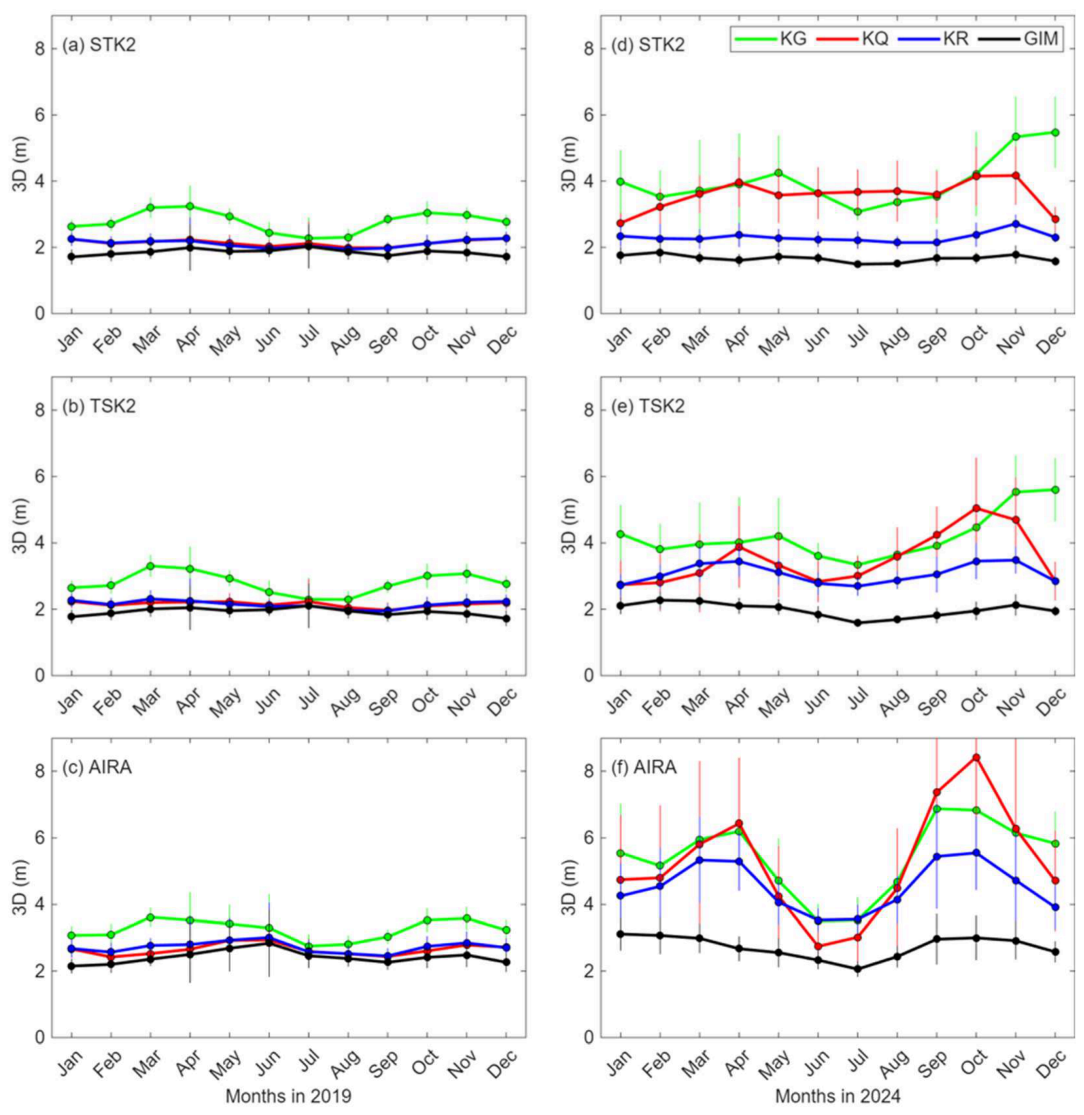
Monthly averaged 3D positioning RMSE derived from 30 s interval SPP results for the solar minimum year (2019, **left column**) and solar maximum year (2024, **right column**). Panels (**a**–**c**) correspond to the STK2, TSK2, and AIRA stations for 2019, and panels (**d**–**f**) correspond to the same stations for 2024, respectively.

Figure 6 presents the monthly mean daily RMSE of three-dimensional positioning errors computed from the SPP results at 30 s intervals, covering a total of 365 days of analysis. The first and second columns of Figure 6 correspond to 2019 and 2024, respectively, and panels (a and d), (b and e), and (c and f) correspond to the STK2, TSK2, and AIRA stations, respectively. In 2019 (solar minimum), the RMSE in all models tended to increase toward lower latitudes. KG showed the largest RMSE across all stations, whereas GIM demonstrated the best performance. KQ and KR generally exhibited comparable levels of performance. Distinct seasonal patterns were observed: three-dimensional errors decreased in summer and winter and increased in spring and autumn owing to the seasonal increase in ionospheric activity around the vernal and autumnal equinoxes [28]. In addition, seasonal patterns varied with station location; the STK2 station at higher latitudes exhibited relatively weak seasonal variation, whereas the AIRA station at lower latitudes showed pronounced seasonal RMSE fluctuations, confirming greater ionospheric variability at lower latitudes.

In 2024 (solar maximum), the RMSE increased substantially across all models compared with that in 2019, reflecting the heightened ionospheric variability associated with increased solar activity. While KQ and KR showed similar performances in 2019, KR generally outperformed KQ by 2024. In particular, at the STK2 station, KR maintained a lower RMSE than KQ throughout all months, and its standard deviation was approximately half that of KQ, demonstrating more stable positioning performance. In contrast, KQ, a model specialized for the Japanese region, performed worse than the global KG model during certain periods (April and September to November), suggesting that KQ has limitations in capturing seasonal ionospheric variations. Furthermore, at the low-latitude AIRA station, there were numerous instances where the vertical error of KQ exceeded that of KG by more than 25 cm. Overall, KR achieved performance comparable to that of KQ during the solar minimum and second only to that of GIM during the solar maximum, confirming improvement over the existing KQ model in terms of both accuracy and stability.

However, the monthly mean results have limitations in fully capturing the fine-grained performance differences that emerge during specific periods. Although KR generally outperformed KQ in 2024, certain periods, approximately two months per year (approximately 16%), including February–March at TSK2 and June–July at AIRA, were identified where KQ yielded a lower RMSE. To analyze this performance reversal in greater detail, the difference in the daily three-dimensional positioning RMSE between KQ and KR was computed.

**Figure 7:**
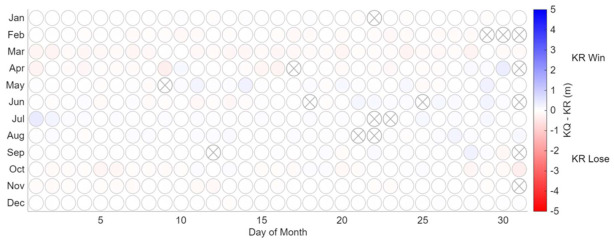
Daily difference in 3D positioning RMSE (KQ − KR) averaged across three GNSS stations (STK2, TSK2, and AIRA) for 2019 (solar minimum). Circles marked with an “X” indicate dates for which data were unavailable. Greater color intensity indicates a larger performance difference between the two models. Blue represents dates on which KR outperformed KQ, whereas red represents dates on which KQ outperformed KR. The predominantly low color intensity reflects the relatively small performance differences observed during the solar minimum period.

Figure 7 presents the monthly distribution of the daily difference in three-dimensional positioning RMSE (KQ − KR), averaged across the three stations for 2019, where positive values indicate that KR outperforms KQ. Among all the days, excluding missing dates, KR was superior on 133 days (37.5%), indicating that KQ was dominant on more days overall. However, the majority of the differences fell within ±0.1 m, confirming that the positioning performance gap between the two models was not substantial. On a monthly basis, negative values were predominant in winter (January–March), indicating a relative advantage for KQ, whereas the light-blue regions in summer (May–August) indicated periods where KR was also superior. These results suggest that during the solar minimum of 2019, the two models showed broadly comparable performances owing to the subdued level of solar activity.

**Figure 8:**
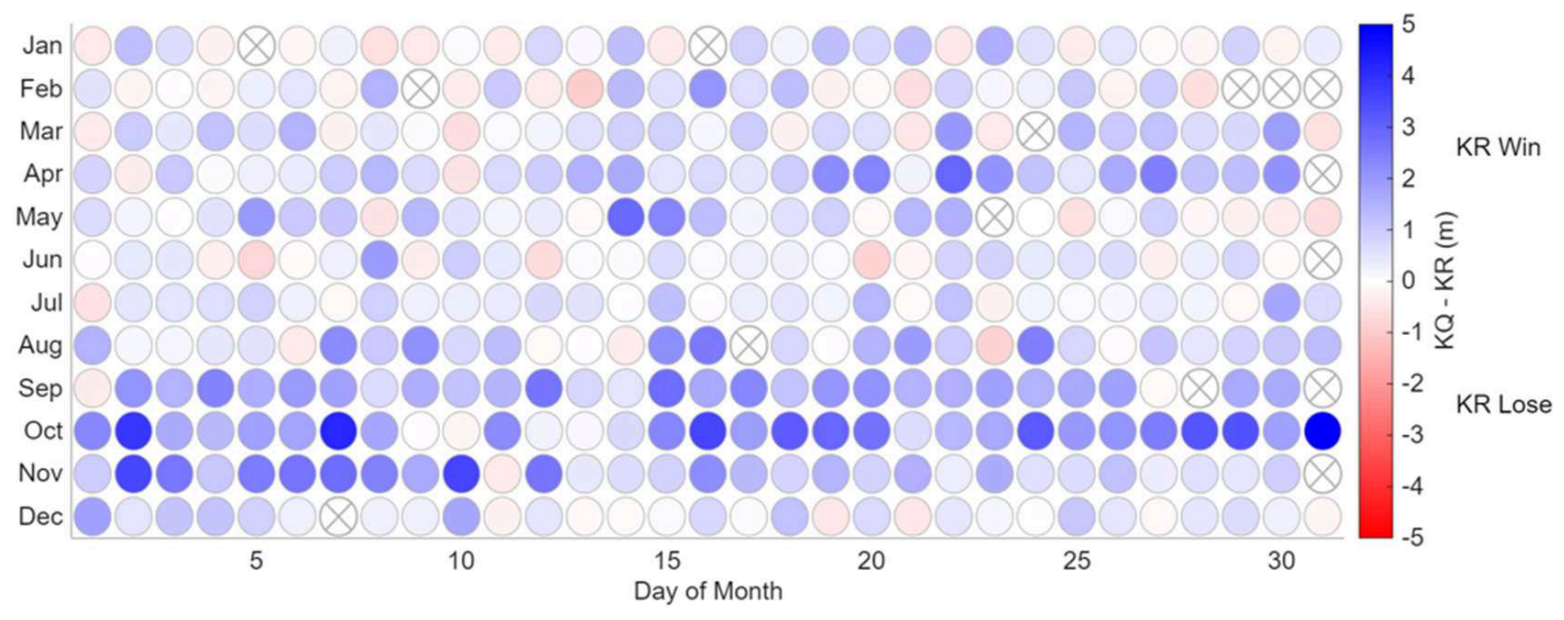
Daily difference in 3D positioning RMSE (KQ − KR) averaged across three GNSS stations (STK2, TSK2, and AIRA) for 2024 (solar maximum). Circles marked with an “X” indicate dates for which data were unavailable. Greater color intensity indicates a larger performance difference between the two models. Blue represents dates on which KR outperformed KQ, whereas red represents dates on which KQ outperformed KR. The predominantly high color intensity reflects the more pronounced performance differences observed during the solar maximum period.

Figure 8 presents the monthly distribution of the daily three-dimensional RMSE difference between KQ and KR, computed in the same manner for 2024. In contrast to 2019, the scale of the differences in 2024 reached a maximum of 4 m, reflecting a substantially enlarged performance gap between the two models; the number of days on which KR was superior increased to 282 days (79.0%), a marked increase from 37.5% in 2019. On a monthly basis, positive values persisted continuously throughout September–November, indicating a clear and consistent advantage for the KR. This is attributed to the inability of KQ to sufficiently capture seasonal ionospheric variations under highly variable ionospheric conditions of the solar maximum, whereas KR more effectively reflected ionospheric characteristics, resulting in improved positioning accuracy. Comparison between 2019 and 2024 confirms that KR maintains performance comparable to that of KQ during the solar minimum, while achieving substantially improved positioning accuracy over KQ during the solar maximum. Consequently, the superior performance of the KR model proposed in this study over existing models was verified through a VTEC-based accuracy evaluation and through actual positioning results.

## 4. Discussion

The Klobuchar model is widely used for single-frequency GNSS ionospheric correction because of its computational simplicity; however, its global coefficients are inherently limited in capturing regional ionospheric characteristics [22]. Although regionally optimized coefficients, such as KQ, have been developed for Japan, comprehensive performance analyses across varying solar activity conditions remain limited. This study applies the methodology proposed by [8] to estimate KR by configuring the grid range to match the target region and evaluating its performance against KG and KQ under both solar minimum and maximum conditions. This approach enabled the estimation of regional coefficients optimized for local users based solely on GIM products, without requiring raw observation data or additional processing. Despite its simplicity, the proposed method outperformed the conventional Klobuchar model and improved the applicability of the Klobuchar model through coefficient estimation tailored to regional ionospheric characteristics.

The results demonstrate that KR provides superior ionospheric correction performance compared with KG and KQ, particularly during solar maximum. During 2024, KR achieved a mean VTEC RMSE that was approximately 50% lower than that of KQ across all latitude bands and outperformed KQ on 79.0% of the valid days in the SPP validation. This result suggests that configuring the grid range to the target region enables the KR to more effectively reflect regional ionospheric variability. Unexpectedly, KQ performed comparably to KR during the solar minimum, with KQ achieving a lower 3D positioning RMSE on 62.5% of the valid days. However, given that differences remained within ±0.1 m for most days, this likely reflects the limited ionospheric variability during solar minimum rather than a fundamental advantage of KQ. Collectively, these findings suggest that KR maintains a performance equivalent to that of KQ during periods of low solar activity, while substantially outperforming KQ during the solar maximum, demonstrating its robustness across varying solar activity conditions.

A key limitation of this study is the use of the IGS final GIM, a post-processing product, for coefficient estimation, which precludes real-time applications. However, the proposed methodology can be extended to real-time operations by substituting the final GIM with a predicted ionospheric model, thereby enabling near-real-time coefficient estimation. Future research incorporating the predicted GIM products will establish KR as a practical real-time ionospheric correction model for single-frequency GNSS applications under varying solar activity conditions.

## 5. Conclusions

In this study, the Klobuchar coefficients optimized for the Japanese region were estimated using IGS GIM data. The analysis region was defined based on the IPP derived from GNSS stations in Japan, and the VTEC values within the defined grid were used as observations to estimate the coefficients using a nonlinear least-squares approach. To account for the varying ionospheric conditions, the solar minimum (2019) and solar maximum (2024) were analyzed, and the temporal characteristics and VTEC RMSE of the coefficients for each period were compared. The results indicate that KR effectively captures the periodic variations associated with solar activity, while maintaining stable performance under both conditions. In particular, the SPP results show that KR achieved a performance comparable to that of KQ during the solar minimum while demonstrating superior accuracy and stability compared with conventional Klobuchar models (KG and KQ) during the solar maximum.

The results of this study demonstrate that the performance of the Klobuchar model can be effectively improved using regionally optimized coefficients rather than the globally broadcast GPS model. The proposed methodology facilitates the straightforward estimation of location-specific coefficients by defining an appropriate grid region tailored to the user’s location, and these findings suggest that estimating Klobuchar coefficients using GIM is an effective approach for enhancing ionospheric correction performance. Notably, compared with the direct use of GIM, the eight-parameter Klobuchar representation requires only simple polynomial computation without spatial interpolation, offering a computationally efficient alternative for regional users.

## Figures and Tables

**Table 1:** Correlation coefficients between Klobuchar models (KG, KQ, and KR) for 2019 (solar minimum).

Model	KG–KQ	KG–KR	KQ–KR
$\alpha_{0}$	0.68	0.79	0.85
$\alpha_{1}$	0.73	0.36	0.44
$\alpha_{2}$	0.05	−0.17	0.05
$\alpha_{3}$	0.25	−0.17	−0.14
$\beta_{0}$	0.07	−0.39	0.35
$\beta_{1}$	0.15	−0.38	0.04
$\beta_{2}$	−0.38	0.46	−0.18
$\beta_{3}$	−0.41	0.63	−0.27

**Table 2:** Correlation coefficients between Klobuchar models (KG, KQ, and KR) for 2024 (solar maximum).

Model	KG–KQ	KG–KR	KQ–KR
$\alpha_{0}$	0.29	0.71	0.25
$\alpha_{1}$	0.17	−0.29	0.09
$\alpha_{2}$	−0.14	0.08	0.06
$\alpha_{3}$	0.10	0.53	0.11
$\beta_{0}$	0.12	0.08	0.10
$\beta_{1}$	0.70	0.50	0.46
$\beta_{2}$	−0.29	0.08	−0.15
$\beta_{3}$	−0.11	0.09	0.04

## Data Availability

The original data used in this study, including the IGS Global Ionospheric Maps (GIM), GNSS observation data from Japanese stations, and GPS/QZSS broadcast ionospheric correction coefficients, are publicly available from the CDDIS archive (https://cddis.nasa.gov/archive/, accessed on 11 June 2026). The solar activity index data are available from the source cited in Reference [21]. The processed datasets generated during the current study are available from the corresponding author upon reasonable request.

## Abbreviations

GNSS	Global Navigation Satellite System
IF	Ionosphere-Free
GIM	Global Ionosphere Map
VTEC	Vertical Total Electron Content
TECU	Total Electron Content Units
GPS	Global positioning system
BDS	BeiDou Navigation Satellite System
QZSS	Quasi-Zenith Satellite System
IGS	International GNSS Service
IPPs	Ionospheric piercing points
SPP	Standard Point Positioning
STEC	Slant Total Electron Content
AMP	Amplitude
PER	Period
IAACs	Ionosphere Associate Analysis Centers
CODE	Center for Orbit Determination in Europe
CAS	Chinese Academy of Sciences
JPL	Jet Propulsion Laboratory
NLSE	Nonlinear least-squares estimation
KASI	Korea Astronomy and Space Science Institute
KQ	Klobuchar QZSS coefficients
KG	Klobuchar GPS coefficients
KR	Klobuchar Regional
RMSE	Root Mean Square Error
EIA	Equatorial Ionization Anomaly
GPT	Global Pressure and Temperature
WLS	Weighted Least-Squares
